# The role of HIF-1 in oncostatin M-dependent metabolic reprogramming of hepatic cells

**DOI:** 10.1186/s40170-016-0141-0

**Published:** 2016-02-17

**Authors:** Nadia Battello, Andreas David Zimmer, Carole Goebel, Xiangyi Dong, Iris Behrmann, Claude Haan, Karsten Hiller, Andre Wegner

**Affiliations:** Luxembourg Centre for Systems Biomedicine, University of Luxembourg, Avenue des Hauts-Fourneaux 7, Esch-Belval, L-4362 Luxembourg; Life Sciences Research Unit, University of Luxembourg, Avenue de la Fäincerie 162a, Luxembourg, 1511 Luxembourg

**Keywords:** Oncostatin M, Inflammation, Stable isotope labeling experiments, Hypoxia-inducible factor, Pyruvate dehydrogenase complex, Pyruvate dehydrogenase kinase

## Abstract

**Background:**

Hypoxia and inflammation have been identified as hallmarks of cancer. A majority of hepatocellular carcinomas are preceded by hepatitis B- or C-related chronic infections suggesting that liver cancer development is promoted by an inflammatory microenvironment. The inflammatory cytokine oncostatin M (OSM) was shown to induce the expression of hypoxia-inducible factor-1 *α* (HIF-1 *α*) under normoxic conditions in hepatocytes and hepatoma cells. HIF-1 *α* is known to orchestrate the expression of numerous genes, many of which code for metabolic enzymes that play key roles in the adaptation of cellular metabolism to low oxygen tension.

**Results:**

Here, we show that OSM-induced upregulation of HIF-1 *α* reprograms cellular metabolism in three clones of the human hepatocyte cell line PH5CH (PH5CH1, PH5CH7, and PH5CH8) towards a hypoxia-like metabolic phenotype but has no significant effect on cellular metabolism of HepG2 and JHH-4 hepatoma cells. Although we observed only minor changes in glucose uptake and lactate secretion in PH5CH8 upon OSM treatment, we identified more pronounced changes in intracellular fluxes based on stable isotope labeling experiments. In particular, glucose oxidation in the tricarboxylic acid (TCA) cycle is reduced through pyruvate dehydrogenase kinase 1 (PDK1)-mediated inhibition of the pyruvate dehydrogenase complex, thereby reducing the oxidative TCA cycle flux. As a result of the impaired mitochondrial glucose and glutamine oxidation, the reductive isocitrate dehydrogenase flux was increased.

**Conclusions:**

We provide evidence that connects the inflammatory mediator OSM to a hypoxia-like metabolic phenotype. In the human hepatocyte cell line PH5CH, OSM-mediated upregulation of HIF-1 *α* and PDK1 can induce hypoxia-like metabolic changes, although to a lesser extent than hypoxia itself. Since PDK1 is overexpressed in several cancers, it might provide a causal link between chronic inflammation and malignant cellular transformation.

**Electronic supplementary material:**

The online version of this article (doi:10.1186/s40170-016-0141-0) contains supplementary material, which is available to authorized users.

## Background

Interleukin 6 (IL6)-type cytokines such as oncostatin M (OSM) are key players in the regulation of the immune response, the immune surveillance of tumor cells, and in the development of cancer [1]. The main functions of IL6-type cytokines are the induction of the acute phase response by the liver, the stimulation of liver regeneration (by inducing hepatocyte proliferation), and the shift from the innate to the adaptive immune response [2, 3]. Aberrant IL6 signaling is likewise able to induce a state of chronic inflammation, as observed in many cancer types and inflammatory diseases. Since hepatocellular carcinoma (HCC) development can be promoted by an inflammatory microenvironment, aberrant IL6 signaling is implicated in the onset of HCC [4, 5]. Elevated IL6 and OSM serum levels in HCC patients have been reported to negatively influence disease outcome [6, 7]. A direct link between IL6 and the onset of HCC was shown in a study by Naugleret et al., where male IL6 knockout mice showed a vastly reduced development of HCC, depicting IL6 as a key mediator in the onset of this cancer [4]. All IL6-type cytokines signal via a homodimer of the transmembrane receptor gp130 or a heterodimer of gp130 with a second receptor chain (e.g., the OSMR or LIFR for OSM), which bind and activate Janus kinases (JAKs) upon cytokine binding. In turn, JAKs activate multiple signaling cascades mainly including STAT3 but also MEK/Erk and the PI3K/Akt pathways [8]. Recently, we have shown in non-neoplastic hepatocytes and HCC cells that STAT3, activated by the IL6-type cytokine OSM, upregulates HIF-1 *α* expression under normoxic conditions, via a transcriptional mechanism, leading to the expression of vascular endothelial growth factor (VEGF) and plasminogen activator inhibitor 1 (PAI) [9]. HIF-1 *α* regulates the expression of numerous target genes, many of which code for metabolic enzymes that play key roles in the adaptation of cellular metabolism to low oxygen tension [10]. For example, HIF-1 *α* promotes high glycolytic rates by upregulating the expression of glucose transporters and many glycolytic enzymes [11]. In addition, pyruvate entry into the citric acid cycle is decreased by HIF-1 *α*-mediated upregulation of pyruvate dehydrogenase (PDH) kinase 1 (PDK1), leading to reduced pyruvate dehydrogenase complex (PDC) activity which results in reduced mitochondrial respiration and increased conversion of pyruvate to lactate [12–14]. Apart from its function in glucose metabolism, a strong impact of HIF-1 *α* on glutamine metabolism has been identified. Resulting from reduced PDC activity, the mitochondrial citrate pool is drastically depleted. Due to these concentration changes, the actual free energy change of the isocitrate dehydrogenase (IDH) reaction will become positive and thus, reversing the IDH flux towards isocitrate. Consequently, glutamine-derived *α*-ketoglutarate is reductively carboxylated to isocitrate by IDH and eventually converted to citrate [15–17]. Malignant cellular transformation and proliferation goes hand-in-hand with reprogrammed cellular metabolism [18, 19], mainly characterized by an increased glucose uptake, a high glycolytic rate, and an increased conversion of pyruvate to lactate, even under normoxic conditions (Warburg effect). Most certainly, this hypoxia-like metabolic phenotype favors increased proliferation rates, as a high glucose turnover provides necessary intermediates such as ribose-5-phosphate, serine, and glycine for macromolecule synthesis. A link between inflammatory STAT3 activity, HIF-1 *α*, and the Warburg effect has been described recently. Demaria and coworkers showed that constitutively active STAT3 induces the Warburg effect in mouse embryonic fibroblasts [20] and renders the cells sensitive for malignant transformation [21]. Based on our previous finding that OSM mediates HIF-1 *α* upregulation through increased mRNA and protein levels in a STAT3-dependent manner, we aimed to investigate the effects of OSM stimulation on central carbon metabolism in hepatocytes and hepatoma cells. Here, we (1) show that OSM induces the expression of HIF-1 *α* in several HCC cell lines and immortalized hepatocytes, (2) demonstrate that OSM stimulation leads to a hypoxia-like metabolic phenotype in three clones of the immortalized hepatocyte cell line PH5CH, (3) provide evidence that HIF-1 *α*-mediated PDK1 upregulation is necessary to induce OSM-dependent metabolic reprogramming in PH5CH cells, and (4) show that OSM-dependent HIF-1 *α* upregulation is not sufficient to induce metabolic reprogramming in the HCC cell lines HepG2 and JHH-4.

## Methods

### Cell culture and reagents

The hepatoma cell lines and the non-neoplastic, SV40 large T antigen-immortalized, hepatocyte lines, PH5CH1, PH5CH7, and PH5CH8 [22] were maintained in Dulbecco’s modified Eagle’s medium (DMEM) (AQMedia, Sigma-Aldrich) supplemented with 10 % fetal calf serum (PAA), 100 mg/l streptomycin, 60 mg/l penicillin, and 25 mM HEPES (Lonza). The SV40 large T antigen-immortalized human liver epithelial cells (THLE-2) were cultured in LHC-8 medium supplemented with 70 ng/ml phosphoethanolamine, 5 ng/ml epidermal growth factor, 10 % FBS, 100 mg/l streptomycin, and 60 mg/l penicillin. Cells were grown at 37 °C in a water-saturated atmosphere at 5 % CO_2_. Hypoxia treatment was performed at 37 °C in a water-saturated atmosphere at 5 % CO_2_ in a hypoxia chamber (C-Chamber (C-274 & C-374) with a ProOx C21 Static O_2_ & CO_2_ Controller from BioSpherix) at the indicated oxygen percentage. HIF-1 *α* and HIF-2 *α* screening experiments were performed in an hypoxia incubator (Heracell) from Thermo Scientific at 37 °C in a water-saturated atmosphere at 5 % CO_2_. Human oncostatin M (227 a.a.) was from PeproTech. For all experiments, cells were seeded together, stimulated for the indicated periods of time, and harvested together at the latest time point.

### Western blot analysis and antibodies

Cells were lysed on the dish with ice-cold lysis buffer containing 30 mM Tris/HCl pH 6.7, 5 % glycerol, 2.5 % mercaptoethanol, and 1 % SDS. Protein extracts were separated by SDS-PAGE and analyzed by Western blotting for the following proteins: HIF-1 *α* and STAT3 (BD Transduction Laboratories), phospho-STAT3, (Cell Signaling), PDH-E1 *α* (pSer232) and PDH-E1 *α* (pSer300) (Merck Millipore), HIF-2 *α* (Novus Biologicals), PDK1 (Enzo Life Sciences) and *α*-tubulin (Thermo Fisher Scientific), and PDH-E1 *α* (pSer293) (Abcam). ECL signals were detected and membranes stripped before re-probing as previously described [23, 24]. The secondary antibodies IRDye 800CW and 680LT (LI-COR Bioscience) were used for fluorescent Western blot detection with the Odyssey Infrared imaging system (LI-COR Biosciences) of PDK1. Quantification of PDK1 protein levels was done using Image Studio Lite version 4.0 (LI-COR Bioscience). For the evaluation the HIF-1 *α* protein levels, densitometric analysis was carried out using the Image-Lab software 4.0 (Bio-Rad Laboratories). The level of the target protein was normalized to the *α*-tubulin protein level.

### Quantitative PCR procedure

Total RNA was extracted using the NucleoSpin RNA II kit (Macherey-Nagel) according to the manufacturer’s instructions. The concentration of isolated RNA was measured using a NanoDrop spectrophotometer. Five hundred nanograms of total RNA was reverse-transcribed with iScript (Bio-Rad Laboratories) in a final volume of 10 *μ*l, according to the manufacturer’s instructions. Quantitative real-time PCR (qPCR) was carried out on an iQ5 Real-Time PCR detection system (Bio-Rad Laboratories). The reaction was performed in a total volume of 10 *μ*l containing cDNA corresponding to 5 ng RNA template, 10 pmol of each forward and reverse primer, and 5 *μ*l iTaq Universal SYBR Green Supermix (Bio-Rad Laboratories). Thermal cycling conditions for all qPCR assays consisted of an initial enzyme activation step at 95 °C for 15 min, followed by 40 cycles of denaturation at 95 °C, and annealing and elongation at 60 °C for 30 s. The housekeeping genes Cyclo A, HPRT, EEF1a, YWHAZ, and the target genes were assayed in parallel for each sample. All samples were run in triplicates. Gene-specific primers for Cyclo A, HPRT, EEF1a, YWHAZ, HIF-1 *α*, HIF-2 *α*, PDK1, and PDP2 were purchased from Eurogentec (Belgium). The geometric mean of three housekeeping genes was calculated, and a normalization factor for each sample was generated using geNorm (VBA add-in for Microsoft Excel). The normalization factor was used to calculate the relative amount of each target in each sample. Each sample was normalized to the untreated control.

Total RNA extraction in HIF-1 *α* silencing experiments was performed using the RNeasy mini kit (QIAGEN) according to the manufacturer’s instructions. Six hundred nanograms of total RNA was reverse-transcribed using SuperScript III reverse transcriptase (Invitrogen) in a final volume of 20 *μ*l according to manufacturer’s recommendations. qPCR was performed on a LightCycler 480 (Roche) in a total volume of 20 *μ*l containing cDNA corresponding to 6 ng RNA template, 5 pmol of each forward and reverse primer, and 10 *μ*l iTaq Universal SYBR Green Supermix (Bio-Rad Laboratories). Cycling conditions were identical to those described earlier. Target gene expression was normalized to the housekeeping gene L27.

### siRNA-mediated silencing of HIF-1 *α* and PDK1

For the siRNA-mediated HIF-1 *α* knockdown, 3 *μ*l of Lipofectamine RNAiMAX (Invitrogen) was diluted in 150 *μ*l 1 × Opti-MEM I (Gibco by Life Technologies) and 1 *μ*l siRNA was added. The final concentration of non-targeting (Dharmacon, Inc.) and HIF-1 *α* siRNA (Santa Cruz) was 15 nM. For the siRNA-mediated PDK1 knockdown, 2.5 *μ*l of Lipofectamine RNAiMAX was diluted in 200 *μ*l 1 × Opti-MEM I and 1 *μ*l siRNA (Dharmacon, Inc.) was added. The final concentration of non-targeting and PDK1 siRNA was 20 nM. The Lipofectamine/siRNA mix was incubated for 20 min at room temperature, then transferred into a 12-well plate and incubated for another 5 min. PH5CH1, PH5CH7, and PH5CH8 cells were directly seeded onto the transfection mix. After 6 h, the medium was changed to complete culture media and cells were grown overnight.

### Stable isotope labeling experiments

Cells were seeded and grown overnight. Complete cell culture medium was then replaced with labeling medium. For isotope labeling experiments using uniformly labeled glucose, cells were cultured in DMEM supplemented with 10 % dialyzed FBS (Invitrogen), 25 mM [^13^C_6_]glucose (Cambridge Isotope Laboratories), and 4 mM glutamine (Sigma-Aldrich). For isotope labeling experiments using uniformly labeled glutamine, cells were grown in DMEM supplemented with 10 % dialyzed FBS (Invitrogen), 25 mM glucose (Sigma-Aldrich), and 4 mM [^13^C_5_] glutamine (Cambridge Isotope Laboratories). Cells were stimulated with 50 ng/ml OSM (PeproTech) and grown for 36 h under normoxia or hypoxia (1 % O_2_) (Jacomex) at 37 °C, 5 % CO_2_ and humidified atmosphere. Metabolites were extracted as described previously [25].

### Extraction of extracellular metabolites

Extracellular metabolites from medium samples were extracted using ice-cold extraction fluid (8:1 methanol/water) containing the internal standard [^13^C_5_]ribitol (Omicron Biochemicals, Inc.) at a concentration of 10 *μ*g/ml. Twenty microliters of the medium was added to 180 *μ*l ice-cold extraction fluid, vortexed for 10 s, and centrifuged at maximum speed for 5 min at 4 °C. Fifty microliters of the medium extracts was transferred to GC-MS glass vials and evaporated under vacuum to dryness at −4 °C using the CentriVap Concentrator.

### GC-MS analysis

Metabolite derivatization was performed using a Gerstel MPS. Dried polar metabolites were dissolved in 15 *μ*l of 2 % methoxyamine hydrochloride in pyridine at 40 °C under shaking. After 60 min, an equal volume of MTBSTFA was added and held for 30 min at 40 °C under continuous shaking. One microliter sample was injected into an SSL injector at 270 °C in splitless mode. GC-MS analysis was performed using an Agilent 7890A GC equipped with a 30-m DB-35MS + 5-m Duraguard capillary column. Helium was used as carrier gas at a flow rate of 1 ml/min. The GC oven temperature was held at 100 °C for 2 min and increased to 300 °C at 10 °C/min. After 3 min, the temperature was increased to 325 °C. The GC was connected to an Agilent 5975C inert XL MSD, operating under electron ionization at 70 eV. The MS source was held at 230 °C and the quadrupole at 150 °C. The detector was operated in single ion mode (see Additional file 1: Table S1 for details). The total run time of one sample was 25.00 min.

### Mass isotopomer distribution analysis

All GC-MS chromatograms were processed using MetaboliteDetector [26]. Chemical formulas for mass isotopomer distribution (MID) determination (Additional file 1: Table S1) were taken from [27]. Weighted carbon contribution was calculated with the following formula: $\frac {1}{n}*\sum \limits _{i=1}^{n} M_{i}*i $, where *n* is the number of carbons of the molecule of interest and *M*_*i*_ the *i*th mass isotopomer.

### Statistical analysis

Error bars represent the standard deviation unless otherwise noted. Statistical significance was determined using two-tailed Student’s *t* test. One asterisk denotes *P*<0.05, two asterisks denote *P*<0.01, and three asterisks denote *P*<0.001.

## Results

### Oncostatin M mediates induction of HIF-1 *α* in hepatocytes and hepatoma cells

**Fig. 1:**
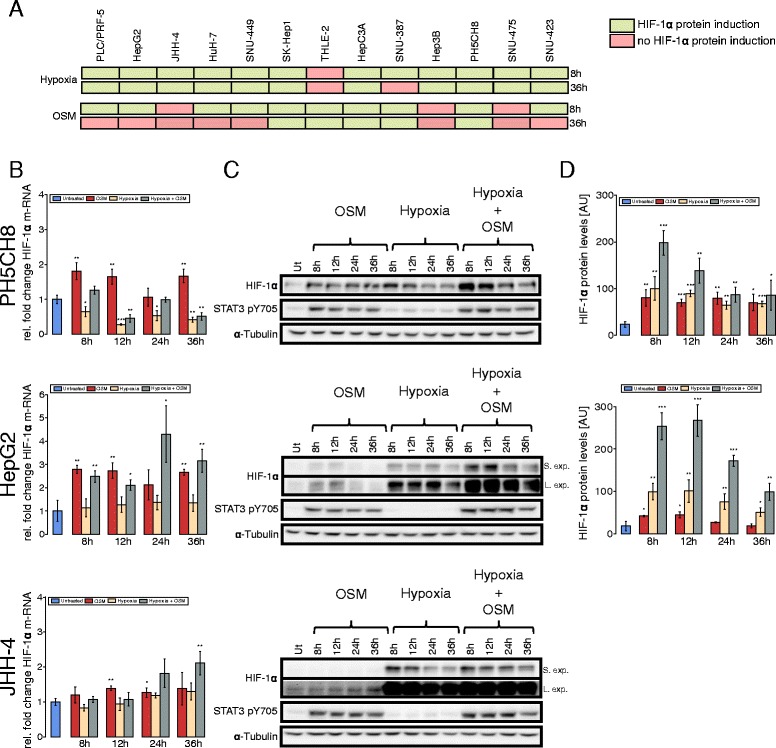
OSM induces HIF expression in hepatoma cells and hepatocytes under normoxic conditions. **a** Eleven HCC cell lines and two non-neoplastic hepatocyte cell lines (THLE-2, PH5CH8) were screened for the induction of HIF-1 *α* protein levels by OSM treatment or hypoxia (*green* indicates protein induction, *red* no HIF-1 *α* protein induction). **b**–**d** HepG2, JHH-4, and PH5CH8 cells were selected for further analysis and treated with 50 ng/ml OSM, hypoxia (1 % O_2_), or a combinatorial treatment for the indicated periods of time. **b** Quantitative RT-PCR of HIF-1 *α*. The fold change was calculated relative to the untreated control. *Error bars* represent the standard deviation of three biological replicates. **c** Western blot analysis of HIF-1 *α*. *α*-Tubulin was used as a loading control, and one representative blot for *α*-tubulin is shown. *S. Exp.* indicates short exposure, *L. Exp.* long exposure. **d** Quantification of HIF-1 *α* protein levels in HepG2 and PH5CH8 cells after the different treatments. Statistical significance was determined in comparison to the untreated control. * *P*<=0.05, ** *P*<=0.01, and *** *P*<=0.001

Based on our previous observation that oncostatin M (OSM) treatment induces HIF-1 *α* expression in hepatocytes under normoxia, we first investigated whether the OSM-mediated upregulation of HIF-1 *α* is a general effect for hepatocytes and hepatoma cells [9]. Therefore, we evaluated HIF-1 *α* protein levels after OSM stimulation and hypoxia in 11 HCC and two non-neoplastic hepatocyte cell lines 8 and 36 h after treatment (Additional file 2: Figure S1). After 8 h, we detected increased HIF-1 *α* protein levels following OSM treatment in eight out of 11 cell lines, whereas after 36 h only five cell lines showed an increase in HIF-1 *α* protein levels (Fig. 1a). Compared to OSM, the effect of hypoxia on HIF-1 *α* protein levels was generally more pronounced (Additional file 2: Figure S1). In ten cell lines, we detected hypoxia-mediated HIF-1 *α* protein induction after 8 h and in nine cell lines after 36 h (Fig. 1a).

We selected three of these cell lines for an in-depth study of OSM-induced effects on cellular metabolism. HepG2 and JHH-4 are hepatocellular carcinoma cell lines, whereas PH5CH8 cells are immortalized hepatocytes. In PH5CH8 and HepG2 cells, we observed that OSM upregulates HIF-1 *α* mRNA expression over the entire time course, while we measured only a weak induction of HIF-1 *α* mRNA expression in JHH-4 cells (Fig. 1b). In non-neoplastic PH5CH8 cells, we observed continuous OSM-dependent induction of HIF-1 *α* protein levels, comparable to those observed under hypoxia (Fig. 1c, d). In HepG2 cells, we detected a transient induction of HIF-1 *α* protein levels, peaking around 12 h post-OSM treatment (Fig. 1c, d). Correlating with the weak induction on HIF-1 *α* mRNA, we did not observed an increase in HIF-1 *α* levels in JHH-4 cells (Fig. 1b, c).

### OSM stimulation reduces glucose oxidation via PDC inhibition under normoxia in non-transformed hepatocytes

**Fig. 2:**
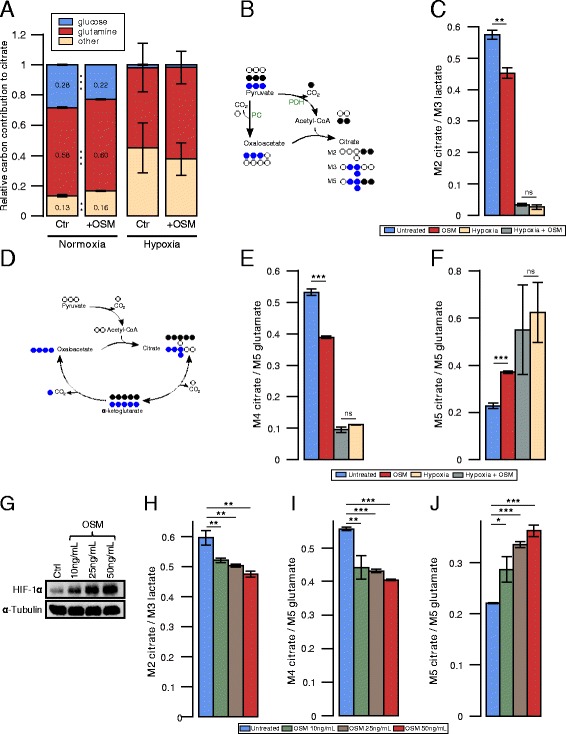
Effect of OSM on central carbon metabolism of PH5CH8 under normoxia and hypoxia. **a**–**f** PH5CH8 cells were treated with 50 ng/ml OSM, hypoxia (1 % O_2_), or a combinatorial treatment, and metabolites were extracted after 36 h. **a** Relative glutamine and glucose carbon contribution to citrate. **b** Carbon atom transitions of pyruvate carboxylase (PC) and pyruvate dehydrogenase complex (PDC). **c** PDC activity determined by the ratio of M2 isotopologues of citrate to M3 isotopologues of lactate, from [^13^C_6_]glucose. **d** Atom transitions for reductive carboxylation and oxidative decarboxylation of *α*-ketoglutarate. **e** Oxidative glutamine contribution to citrate, determined by the ratio of M4 isotopologues of citrate to M5 isotopologues of glutamate, from [^13^C_5_]glutamine. **f** Reductive glutamine contribution to citrate, determined by the ratio of M5 isotopologues of citrate to M5 isotopologues of glutamate, from [^13^C_5_]glutamine. **g**–**j** PH5CH8 cells were treated with different concentrations of OSM. Metabolites and proteins were extracted after 36 h. **g** HIF-1 *α* protein levels in PH5CH8 cells after treatment with the indicated OSM concentrations. **h** PDC activity determined by the ratio of M2 isotopologues of citrate to M3 isotopologues of lactate, from [^13^C_6_]glucose. **i** Oxidative glutamine contribution to citrate, determined by the ratio of M4 isotopologues of citrate to M5 isotopologues of glutamate, from [^13^C_5_]glutamine. **j** Reductive glutamine contribution to citrate, determined by the ratio of M5 isotopologues of citrate to M5 isotopologues of glutamate, from [^13^C_5_]glutamine. All *error bars* indicate the standard deviation. All *p* values and error bars are calculated from at least two independent replicates (*n*>=2). Statistical significance was determined in comparison to the untreated control. * *P*<=0.05, ** *P*<=0.01, and *** *P*<=0.001

Based on the observed OSM-induced upregulation of HIF-1 *α* under normoxia, we hypothesized that this would induce a pseudo-hypoxic metabolic phenotype. To investigate the effect of OSM stimulation on central carbon metabolism, we performed stable isotope labeling experiments using [^13^C_6_]glucose and [^13^C_5_]glutamine and quantified the isotopic enrichment in form of MIDs. Therefore, we cultured PH5CH8 cells under normoxia (18.6 % O_2_), hypoxia (1 % O_2_) either with or without 50 ng/ml OSM. We observed a significant decrease (*P*<0.001) in glucose carbon contribution to citrate in OSM-stimulated cells under normoxia as compared to the control (Fig. 2a), although glucose uptake was slightly increased (Additional file 3: Figure S2A). In line with a reduced glucose carbon contribution to the TCA cycle, we observed increased lactate secretion (Additional file 3: Figure S2A). We next investigated whether the decreased glucose contribution is a result of reduced anaplerosis via pyruvate carboxylase (PC) or reduced PDC activity. Using [^13^C_6_]glucose as a tracer, the two pathways can be distinguished by analyzing the M2 and M3 isotopologues of citrate. Briefly, PDC activity generates [^13^C_2_]acetyl-CoA from which M2 citrate is produced, whereas PC activity yields [^13^C_3_]oxaloacetate leading to M3 citrate (Fig. 2b). M5 isotopologues of citrate reflect the combined carbon contribution of PC and PDC to citrate. To exclude bias from the overall enrichment, we normalized the abundance of the M2, M3, and M5 isotopologues of citrate to the abundance of the M3 isotopologue of lactate. While there was no difference in M3 and M5 isotopologues (data not shown), OSM-stimulated cells exhibited a significantly (*P*=0.003) reduced M2-citrate-to-M3-lactate ratio under normoxic conditions, pointing to reduced glucose catabolism via PDC (Fig. 2c) and thus a reduced oxidative TCA cycle flux. Reduced M2 isotopologues propagated to all analyzed downstream metabolites in the TCA cycle (Additional file 4: Table S2). Although HIF-1 *α* protein levels upon OSM treatment were comparable to those under hypoxic conditions (Fig. 1d), we observed a much stronger effect on cellular metabolism induced by hypoxia. Specifically, PDC activity and consequently glucose carbon contribution to citrate were almost reduced to zero (Fig. 2a, c). OSM treatment did not further impact glucose oxidation under hypoxia (Fig. 2a, c).

**Fig. 3:**
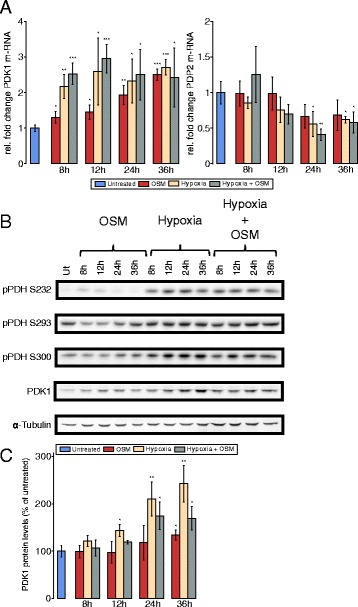
PDK1 levels are increased upon OSM stimulation under normoxia in PH5CH8 non-neoplastic hepatocytes. PH5CH8 cells were treated with OSM (50 ng/ml), hypoxia (1 % O_2_), or a combinatorial treatment for the indicated periods of time. **a** Quantitative RT-PCR of PDK1 and PDP2 mRNA. The fold change was calculated relative to the untreated control. **b** Western blot analysis for all three PDH phosphorylation sites (S232, S293, S300) and PDK1. *α*-Tubulin was used as a loading control, and one representative *α*-tubulin blot is shown. **c** Quantification of PDK1 protein levels, shown as percentage of the untreated control. All *error bars* indicate the standard deviation from three biological replicates. Statistical significance was determined in comparison to the untreated control. * *P*<=0.05, ** *P*<=0.01, and *** *P*<=0.001

Since PDC links glycolysis and TCA cycle, it is tightly regulated. Its activity is mainly controlled through reversible phosphorylation of the PDH-E1 *α* subunit. An inhibition of the complex is mediated by its phosphorylation via pyruvate dehydrogenase kinases (PDK1-4), whereas a dephosphorylation through pyruvate dehydrogenase phosphatases (PDP1-2) leads to an increase in PDC activity. PDK1 is a direct HIF-1 *α* target gene, and its upregulation has been implicated in the metabolic switch induced by HIF-1 *α* [13]. For that reason, we determined the effect of OSM treatment on PDK1 and PDP2 mRNA levels. Furthermore, we analyzed the degree of phosphorylation of all three serine phosphorylation sites of the PDH-E1 *α* subunit by Western blot. We observed an increase in the expression of PDK1 following OSM treatment, which was, however, weaker compared to hypoxia and hypoxia in combination with OSM treatment (Fig. 3a–c). The transcription of PDP2 was decreased under hypoxia at later time points but remained unaffected by OSM (Fig. 3a). Moreover, hypoxia led to increased phosphorylation levels of the PDH-E1 *α* subunit at all three serine residues, whereas the results for OSM were not as unambiguous. We did not observe a clear effect on any of the three serine residues after OSM treatment (Fig. 3b). Hypoxia led to higher PDK1 protein levels than OSM, correlating with the weaker effect of OSM on PDC activity. Collectively, these data indicate that OSM treatment reduces PDC activity under normoxia in PH5CH8 cells but to a weaker extent than hypoxia.

### OSM stimulation increases reductive carboxylation of *α*-ketoglutarate

Because of the observed reduced glucose oxidation in the TCA cycle via PDC inhibition, we next sought to investigate whether this reduction is compensated by an increased glutamine contribution. To quantify the relative contribution of glutamine to the TCA cycle, we cultured PH5CH8 cells in the presence of [^13^C_5_]glutamine. We observed a modest increase of glutamine-derived carbon in citrate under OSM-stimulation (Fig. 2a). Using [^13^C_5_]glutamine as a tracer, the M4 isotopologue of citrate results from a condensation of [^13^C_4_]oxaloacetate and unlabeled acetyl-CoA (Fig. 2d), thereby providing a readout of relative glutamine oxidation in the TCA cycle. Although the fractional carbon contribution of glutamine to citrate was increased, we observed a decreased relative M4 isotopologue abundance when stimulating PH5CH8 cells with OSM under normoxic conditions (Fig. 2e), pointing to a decreased glutamine oxidation in the TCA cycle upon OSM stimulation. This result suggests that the glucose-derived acetyl-CoA pool is not fully replenished by an alternative acetyl-CoA source such as increased fatty acid oxidation or degradation of branched chain amino acids. We also observed reduced M4 isotopologues in downstream metabolites including malate, fumarate, and aspartate (Additional file 5: Table S3). Alternatively, citrate can be generated through reductive carboxylation of *α*-ketoglutarate via IDH, eventually yielding M5 citrate (Fig. 2d). Reductive carboxylation was significantly increased (*P*<0.001) in OSM-stimulated cells compared to the control, as indicated by an increased M5 isotopologue abundance (Fig. 2f). In agreement with an increased relative reductive IDH flux, we observed increased *α*-ketoglutarate levels together with decreased citrate levels in OSM-stimulated cells (see Additional file 3: Figure S2B). In line with the higher reduction of PDC activity under hypoxia (Fig. 2c), we observed a much stronger suppression of glutamine oxidation (Fig. 2e) and a higher induction of reductive IDH activity under hypoxia (Fig. 2f). However, OSM treatment did not have any effect on glutamine metabolism under hypoxia (Fig. 2e, f).

### OSM stimulation reduces glucose oxidation and increases reductive glutamine metabolism in a concentration-dependent manner

To investigate whether OSM affects cellular metabolism in a dose-dependent manner, we cultured PH5CH8 cells with 10, 25, and 50 ng/ml OSM. We found that OSM-mediated HIF-1 *α* protein induction steadily increased with the OSM concentration (Fig. 2g). While we observed the strongest effect on cellular metabolism with 50 ng/ml OSM, we observed a reduction of glucose and glutamine oxidation as well as increase of reductive glutamine metabolism already with 10 ng/ml OSM (Fig. 2h–j).

### HIF-1 *α*-mediated PDK1 upregulation is critical for OSM-induced metabolic reprogramming in PH5CH8 cells

**Fig. 4:**
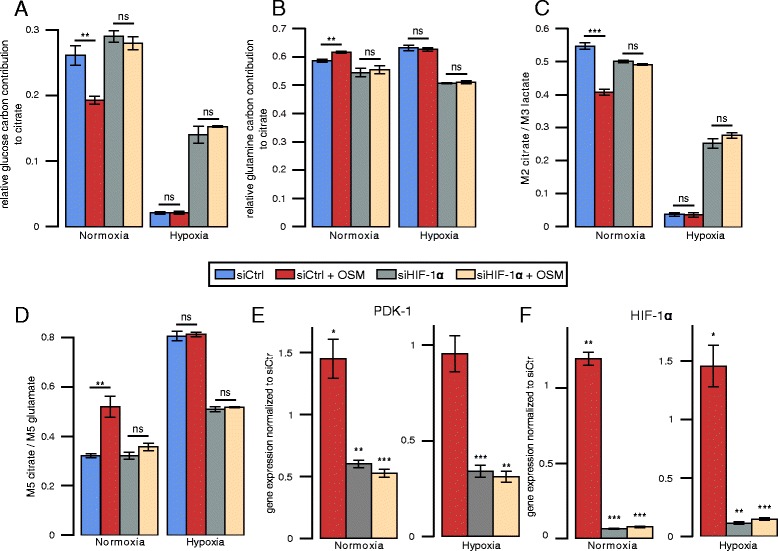
Metabolic effects of HIF-1 *α* silencing on the OSM-induced metabolic phenotype. **a** Relative glucose contribution to citrate. **b** Relative glutamine contribution to citrate. **c** PDH activity determined by the ratio of M2 isotopologues of citrate to M3 isotopologues of lactate, from [^13^C_6_]glucose. **d** Reductive glutamine contribution to citrate, determined by the ratio of M5 isotopologues of citrate to M5 isotopologues of glutamate, from [^13^C_5_]glutamine. **e** PDK1 gene expression. **f** HIF-1 *α* gene expression. Fold changes for qPCRs were calculated relative to the gene expression in control samples under normoxia or hypoxia, respectively. All *error bars* indicate the standard deviation. All *p* values and error bars are calculated from at least two independent replicates (*n*>=2). Metabolites were extracted after 36 h of indicated treatment. Statistical significance was determined in comparison to the untreated control. * *P*<=0.05, ** *P*<=0.01, and *** *P*<=0.001

To validate the hypothesis that OSM-induced metabolic reprogramming is caused by increased HIF-1 *α* activity, we silenced HIF-1 *α* expression in PH5CH8 using siRNA (Fig. 4f). A scrambled siRNA was included as a control to exclude secondary effects of the transfection. Silencing of HIF-1 *α* almost completely prevented the metabolic reprogramming induced by OSM. Specifically, glucose-derived carbon in citrate increased under normoxia and hypoxia (Fig. 4a) and was comparable to levels of untreated cells (Fig. 2a). Glutamine carbon contribution to citrate decreased in HIF-1 *α*-silenced cells under normoxia and hypoxia (Fig. 4b). Moreover, silencing of HIF-1 *α* decreased PDK1 expression (Fig. 4e), increased PDC activity (Fig. 4c), and decreased reductive carboxylation (Fig. 4d). Overall, these data suggests that HIF-1 *α* is critical for OSM-dependent metabolic phenotype observed in PH5CH8 cells.

**Fig. 5:**
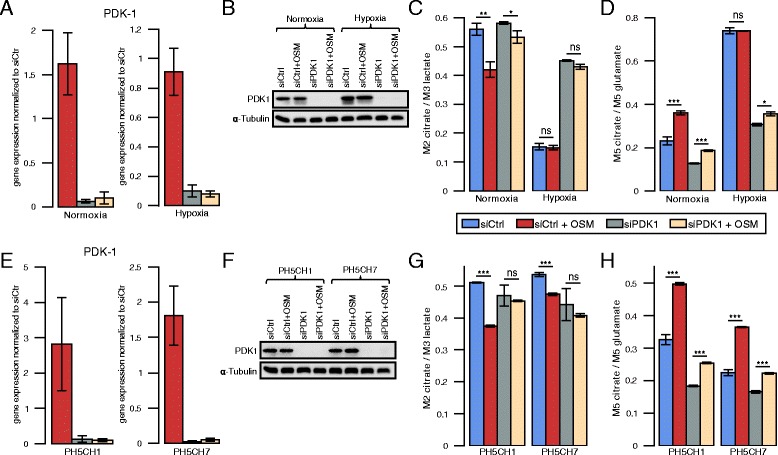
Metabolic effects of PDK1 silencing on the OSM-induced metabolic phenotype in three PH5CH hepatocyte clones. **a** PDK1 mRNA expression in PH5CH8 cells. Fold changes were calculated relative to PDK1 expression in control samples under normoxia or hypoxia, respectively. **b** PDK1 protein expression in PH5CH8 cells. **c** PDH activity in PH5CH8 cells determined by the ratio of M2 isotopologues of citrate to M3 isotopologues of lactate, from [^13^C_6_]glucose. **d** Reductive glutamine contribution to citrate in PH5CH8 immortalized hepatocytes, determined by the ratio of M5 isotopologues of citrate to M5 isotopologues of glutamate, from [^13^C_5_]glutamine. **e** PDK1 mRNA and **f** protein levels in PH5CH1 and PH5CH7 cells under normoxia. **g** PDH activity under normoxia in PH5CH1 and PH5CH7 cells determined by the ratio of M2 isotopologues of citrate to M3 isotopologues of lactate, from [^13^C_6_]glucose. **h** Reductive glutamine contribution to citrate under normoxia in PH5CH1 and PH5CH7 immortalized hepatocytes, determined by the ratio of M5 isotopologues of citrate to M5 isotopologues of glutamate, from [^13^C_5_]glutamine. Metabolites were extracted after 36 h of indicated treatment. All *error bars* indicate the standard deviation. All *p* values and error bars are calculated from at least three independent replicates (*n*>=3). Statistical significance was determined in comparison to the untreated control. * *P*<=0.05, ** *P*<=0.01, and *** *P*<=0.001

Based on the observation that PDK1 expression is increased by OSM treatment in an HIF-1 *α*-dependent manner, we silenced PDK1 in PH5CH8 cells (Fig. 5a, b). As a consequence of suppressed PDK1 expression, previously observed OSM-mediated metabolic changes were attenuated. Both the OSM-induced changes in PDC activity and reductive carboxylation of *α*-ketoglutarate were impaired when PDK1 was silenced (Fig. 5c, d). Under hypoxic conditions, we detected a pronounced increase in PDC activity and drastically reduced reductive glutamine metabolism (Fig. 5c, d). Taken together, our results show that the OSM-induced metabolic phenotype in PH5CH8 cells is mediated by HIF-1 *α*-dependent PDK1 upregulation.

### OSM induces PDK1-dependent metabolic reprogramming in other PH5CH clones

In order to show that OSM-mediated metabolic reprogramming is not a unique feature in PH5CH8 cells, we aimed to validate our main findings in two other PH5CH clones PH5CH1 and PH5CH7. In line with our findings in PH5CH8, we detected continuous OSM-dependent HIF-1 *α* and PDK1 mRNA upregulation and protein induction (Additional file 6: Figure S3A and S3D), while PDP2 mRNA levels were downregulated. HIF-1 *α* protein levels were comparable to those found under hypoxia (Additional file 6: Figure S3B and S3E). Furthermore, OSM treatment reduced the entry of glucose-derived carbon into the TCA cycle and induced reductive glutamine metabolism (Fig. 5g, h). As already observed in PH5CH8, silencing of PDK1 attenuated the OSM-mediated metabolic changes in PH5CH1 and PH5CH7 cells (Fig. 5g, h). Taken together, these results confirm that PDK1 is required to induce a hypoxia-like metabolic phenotype in PH5CH1 and PH5CH7 cells.

### OSM-dependent HIF-1 *α* upregulation is not sufficient to induce a hypoxia-like metabolic phenotype in two hepatoma cell lines

**Fig. 6:**
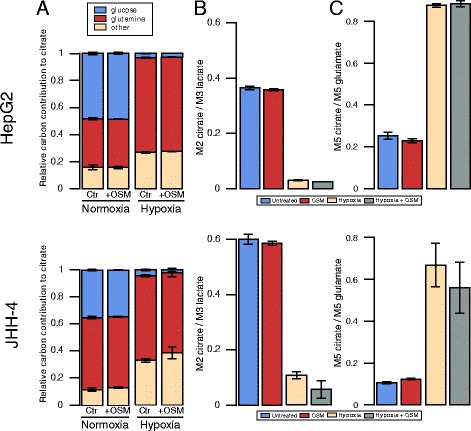
Effect of OSM treatment on central carbon metabolism of HepG2 and JHH-4. **a** Relative carbon contribution to citrate. **b** PDC activity determined by the ratio of M2 isotopologues of citrate to M3 isotopologues of lactate, from [^13^C_6_]glucose. **c** Reductive glutamine contribution to citrate, determined by the ratio of M5 isotopologues of citrate to M5 isotopologues of glutamate, from [^13^C_5_]glutamine. All *error bars* indicate the standard deviation. All error bars are calculated from at least two independent replicates (*n*>=2). Metabolites were extracted after 36 h of indicated treatment

**Fig. 7:**
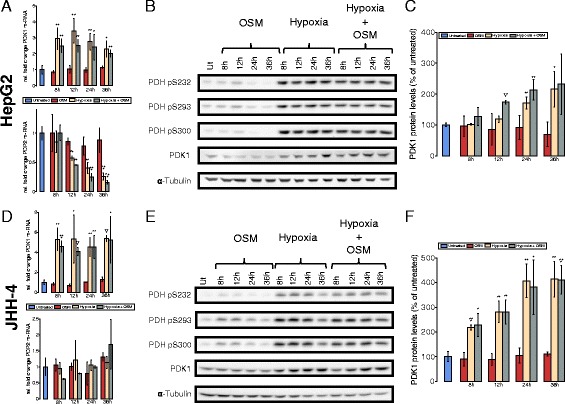
OSM does not influence PDK1 levels in the two HCC cell lines. Cells were treated with OSM (50 ng/ml), hypoxia 1 % O_2_, a combinatorial treatment for the given time points. **a**, **d** qRT-PCR of PDK1 and PDP2 after the indicated period of time and treatment, mRNA from three biological replicates in HepG2 (**a**) and JHH-4 (**d**) cells. Fold change was calculated to the untreated control. **b**, **e** Western blot analysis of all three PDH phosphorylation sites (S232, S293, S300) and PDK1 in HepG2 (**b**) and JHH-4 (**d**) cells. *α*-Tubulin was used as a loading control and one representative *α*-tubulin blot is shown. **c**, **f** PDK1 protein levels in HepG2 (**c**) and JHH-4 (**f**) cells after the indicated period of time and treatment, shown as percentage of the untreated control. All *error bars* indicate the standard deviation calculated from three biological replicates. Statistical significance was determined in comparison to the untreated control. * *P*<=0.05, ** *P*<=0.01, and *** *P*<=0.001

Since aberrant IL6 signaling is implicated in the onset of HCC development, we next sought to investigate the effect of OSM on the two HCC cell lines HepG2 and JHH-4. Although HIF-1 *α* mRNA was increased in HepG2 (Fig. 1b) and HIF-1 *α* protein was transiently induced by OSM treatment (Fig. 1c, d), we did not observe changes in glucose and glutamine metabolism after 36 h of OSM stimulation (Fig. 6a–c). To verify that OSM has indeed no metabolic effect, we profiled cellular metabolism of HepG2 at 6 and 15 h post-OSM stimulation (Additional file 7: Figure S4). In line with HIF-1 *α* mRNA and protein expression levels in JHH-4 cells (Fig. 1b, c), we did not observe OSM-mediated metabolic changes (Fig. 6a–c). Under hypoxic conditions, however, glucose oxidation was suppressed, while glutamine carbon contribution was highly increased in both cell lines (Fig. 6a). In line with these data, we observed decreased PDC activity under hypoxia but no effect of OSM treatment under normoxia (Fig. 6b). We observed the same for reductive carboxylation of *α*-ketoglutarate, which was increased under hypoxia but not significantly affected by OSM treatment under normoxia (Fig. 6c). In agreement with the MID data, we did not observe an induction of PDK1 (Fig. 7a, c, d, f). Concordantly, phosphorylation at any of the three serine residues of PDH was not induced after OSM stimulation under normoxia in these two cell lines (Fig. 7b, e). Taken together, these data show that, in HepG2 and JHH-4, OSM did not induce the metabolic changes observed in the three clones of the human hepatocyte cell line PH5CH.

## Discussion

Along with others, we reported that transcription of HIF-1 *α*, one of the master regulators of the cellular response to hypoxia, is upregulated through IL6-type cytokine (e.g., IL6 and OSM) signaling or other activators of the JAK/STAT3 signaling pathway [9, 20]. Because HIF-1 *α* is heavily induced by hypoxia, we hypothesized that OSM induces a hypoxia-like metabolic phenotype under normoxic conditions. In this study, we provide insights in how OSM affects transcriptional regulation of central metabolism and how the transcriptional changes propagate to redirect intracellular metabolic fluxes. First, we demonstrated that OSM treatment under normoxia leads to an upregulation of PDK1 in three clones of the immortalized hepatocyte cell line PH5CH. Increased mRNA levels of PDK1 translated to increased protein levels (Fig. 3a, c; Additional file 6: Figure S3C), which correlated with reduced PDC activity (Fig. 2c, Fig. 5g) and therefore reduced glucose oxidation in the TCA cycle. Reduced glucose-derived acetyl-CoA was not compensated by an alternative acetyl-CoA source such as fatty acid oxidation or degradation of branched chain amino acids, leading to reduced glutamine oxidation (Fig. 2e, Fig. 5h). As a result of the reduced glucose and glutamine oxidation, the intracellular citrate levels decreased, triggering reductive *α*-ketoglutarate carboxylation by mass action [15, 28]. This is in line with a previous study by Gameiro et al., showing that reductive carboxylation is triggered by a deficient mitochondrial pyruvate oxidation [16]. These results indicate that PDK1 plays an important role in the OSM-induced metabolic phenotype. Together, our current work shows that OSM treatment under normoxic conditions induces an intermediate metabolic phenotype between hypoxia (1 % O_2_) and normoxia (18.6 % O_2_) in PH5CH8 cells. This metabolic phenotype can support high proliferation rates. Although OSM was implicated in increased hepatocyte proliferation in vivo after partial hepatectomy (Nakamura et al. 2004), we did not observe an effect on the growth rate of PH5CH8 cells (data not shown).

Second, to confirm our hypothesis that the OSM-induced metabolic changes are HIF-1 *α*-dependent, we demonstrated that silencing of HIF-1 *α* almost completely attenuated the OSM-dependent metabolic effects under normoxia, showing that HIF-1 *α* expression is critical for OSM-dependent metabolic reprogramming. This dependency on HIF-1 *α* activity explains why although OSM leads to increased HIF-1 *α* protein levels under hypoxia, it does not have any additional effect, as with 1 % oxygen, the amount of transcriptionally active HIF-1 *α* cannot be further induced through OSM. Interestingly, silencing of HIF-1 *α* under hypoxia increased glucose-derived pyruvate oxidation, suggesting that glucose-derived acetyl-CoA and thus downregulated PDC activity is the limiting factor and not only reduced O_2_ availability for respiration under hypoxia.

Third, we identified PDK1 as a critical HIF-1 *α* downstream regulator of the OSM-induced metabolic changes. PDK1 knockdown, however, did not fully abolish OSM-mediated effects on intracellular metabolic fluxes (Fig. 5c, d, g, h). Possibly, our siRNA-mediated PDK1 silencing was not sufficiently strong to fully abolish PDK1 activity, although PDK1 protein levels were not detectable by Western blot anymore (Fig. 5b, f). Furthermore, it cannot be excluded that yet another regulator is involved in the mediation of OSM-dependent metabolic reprogramming.

Finally, we demonstrated that in HepG2 and JHH-4, OSM did not induce the metabolic changes we observed in the PH5CH clones (Figs. 6 and 7). This is potentially explained by the fact that OSM did not induce HIF-1 *α* in JHH-4 or solely in a transient way in HepG2. Even in the PH5CH clones, where OSM-induced HIF-1 *α* protein levels are similar to those induced by hypoxia, the impact on PDK1 expression was less pronounced. In addition, the metabolic effect observed upon OSM in the PH5CH clones was lower compared to the metabolic changes induced by hypoxia. Taken together, these data raise the hypothesis that OSM-induced HIF-1 *α* is not as potent in activating HIF downstream responses as hypoxic HIF. This might be due to the fact that HIF-1 *α* is post-translationally regulated by the following mechanisms: (1) Under normoxia, OSM-induced HIF-1 *α* might still be hydroxylated by PHDs on proline and asparagine residues. Hence, VHL binds HIF-1 *α* and tags it for proteasomal degradation leading to a faster degradation as compared to stabilized HIF-1 *α* under hypoxia. VHL binding complexes might also interfere with HIF-mediated transcription or lead to a reduced binding time of HIF on promotors. In addition, factor-inhibiting HIF (FIH)-mediated asparagine hydroxylation in the transactivation domain of HIF-1 *α* prevents cofactor binding (CEBP) and might thereby reduce effective transcription [29, 30]. (2) HIF-1 *α* might be modified by other post-translational modifications upon hypoxia compared to OSM-induced HIF-1 *α* under normoxia and thus altering its function. In addition to hydroxylation, HIF1 *α* is known to undergo acetylation, phosphorylation, S-nitrosylation, and SUMOylation [31]. (3) Known cofactors of HIF-1 *α* (e.g., TAZ) found under hypoxia might not be present in normoxic conditions [32].

We have previously shown, however, that at least the HIF-1 *α* target genes VEGF and PAI as well as HIF-1 *α*-dependent reporter gene constructs under the control of the VEGF or the PAI promoter or of HRE elements are upregulated upon OSM stimulation [9]. Hence, OSM-induced HIF-1 *α* can be an active transcription factor in HepG2 cells. However, it remains unclear why we were not able to detect HIF-1 *α*-induced metabolic changes in HepG2 cells in this study. VEGF was discussed to be not only a HIF-1 *α* but also a STAT3 target gene [33]. Therefore, it is possible that VEGF and PAI are regulated by both transcription factors. In contrast to HIF, STAT3 might be able to recruit cofactors under normoxia that HIF cannot, as it remains hydroxylated at asparagine. For many metabolic genes (which are not STAT3 responsive or mainly rely on HIF-1 *α* for transcriptional regulation), asparagine hydroxylation might prevent HIF from being fully active.

Collectively, our data shows that HIF-1 *α* is necessary but not sufficient to induce OSM-dependent metabolic reprogramming of hepatic cells.

## Conclusions

Hypoxia and inflammation have been recognized as conditions favoring carcinogenesis [18]. Both microenvironments induce profound changes in cellular metabolism towards a more glycolytic phenotype [34], and it is assumed that high rates of glycolysis provide a selective growth advantage that promotes tumor progression [35]. However, it is still debated whether these metabolic changes are a consequence or a cause of carcinogenesis [35]. The OSM-induced metabolic changes described in this study would support increased proliferation rates and are similar to the metabolic adaptations described in tumors [19]. Hence, OSM-dependent metabolic reprogramming might serve as a first hit in carcinogenesis, which connects chronic infections to increased risks of tumor development. On the other hand, our experiments only applied acute treatments (50 ng/ml OSM, 18.6 % O_2_, and 1 % O_2_) and certainly do not reflect chronic conditions as encountered in a tumor. It will be interesting to investigate whether long-term activity of the JAK/STAT3 pathway affects hepatocyte/hepatoma cell metabolism in a different way.

## Additional files

Additional file 1
**Table S1.** For GC/MS measurements, the detector was operated in single ion mode (SIM). Metabolite specific parameters (selected ions, dwell time, retention time) are described. (PDF 66.8 kb)

Additional file 2
**Figure S1.** Effects of OSM and hypoxia on HIF-1 *α* and HIF-2 *α* protein expression in hepatoma cells and hepatocytes. Eleven hepatoma cell lines and two immortalized hepatocyte cell lines (PH5CH8 and THLE-2) were treated with 20 ng/ml OSM (O) or grown at 1 % O_2_ (H) over 8 and 36 h. (A) HIF-1 *α* protein expression after OSM treatment represented in comparison to untreated cells (Ut). (B) HIF-1 *α* protein expression after OSM treatment represented in comparison to cells grown under hypoxia. (C) HIF-2 *α* protein expression after OSM treatment represented in comparison to untreated cells. (D) HIF-2 *α* protein expression after OSM treatment represented in comparison to cells grown under hypoxia. (E) HIF-1 *α* and HIF-2 *α* protein induction in non-neoplastic THLE-2 hepatocytes after OSM or hypoxia or a combinatorial treatment. (PDF 655 kb)

Additional file 3
**Figure S2.** (A) Glucose uptake and lactate secretion rates in PH5CH8 immortalized hepatocytes in response to a 36 h treatment with 50 ng/mL OSM. Uptake and secretion rates were determined by GC/MS. (B) *α*-ketoglutarate to citrate ratios in PH5CH8 cells treated with 50ng/mL of OSM for 36 h in comparison to the respective untreated control. (PDF 42.2 kb)

Additional file 4
**Table S2.** Mass isotopomer distributions (MIDs) from [^13^C_6_]glucose in PH5CH8 immortalized human hepatocytes treated for 36 h with 50 ng/mL OSM or left untreated. MIDs were corrected for natural isotope abundance. (PDF 94.2 kb)

Additional file 5
**Table S3.** Mass isotopomer distributions (MIDs) from [^13^C_5_]glutamine in PH5CH8 immortalized human hepatocytes treated for 36 h with 50 ng/mL OSM or left untreated. MIDs were corrected for natural isotope abundance. (PDF 94.2 kb)

Additional file 6
**Figure S3.** Response of PH5CH1 and PH5CH7 non-neoplastic hepatocytes to OSM, hypoxia, and a combination of both stimuli. PH5CH1 and PH5CH7 cells were treated with OSM (50 ng/ml), hypoxia (1 % O_2_), or a combinatorial treatment for the indicated periods of time. (A, D) Quantitative RT-PCR of PDP2, PDK1, and HIF-1 *α* mRNA. Fold changes were calculated relative to the untreated control. (B, E) Western blot analysis for HIF-1 *α*, HIF-2 *α*, PDK1, and all three PDH phosphorylation sites (S232, S293, S300). Vinculin and *α*-tubulin were used as loading controls, and one representative blot is shown. (C, F) Quantification of PDK1 protein levels. (PDF 428 kb)

Additional file 7
**Figure S4.** Mass isotopomer distributions (MIDs) from [^13^C_6_]glucose in HepG2 human hepatoma cells treated for 6 and 15 h with 50 ng/mL OSM or left untreated. MIDs were corrected for natural isotope abundance. (PNG 14.8 kb)
