# Supplementary material for: The role of HIF-1 in oncostatin M-dependent metabolic reprogramming of hepatic cells
Source: Cancer Metab. 2016 Feb 17;4:3. doi: 10.1186/s40170-016-0141-0 (PMC4756539; doi:10.1186/s40170-016-0141-0)
Supplement: Additional file 1 — Table S1. For GC/MS measurements, the detector was operated in single ion mode (SIM). Metabolite specific parameters (selected ions, dwell time, retention time) are described. (PDF 66.8 kb) [file 40170_2016_141_MOESM1_ESM.pdf]

Additional file 1: Table S1

Compound	Ions	Exact ion	dwell time (ms)	Sum formula	RT (min)
Pyruvic acid 1MeOX 1TBDMS	174–180	0	15	$C_6H_{12}O_3NSi$	6.62
Lactic acid 2TBDMS	261–267	0.1	15	$C_{11}H_{25}O_3Si_2$	8.35
Alanine 2TBDMS	260–266	0.1	15	$C_{11}H_{26}NO_2Si_2$	8.89
Glycine 2TBDMS	246–252	0.1	15	$C_{10}H_{24}NO_2Si_2$	9.4
γ-Aminobutyric acid 2TBDMS	274–281	0.2	15	$C_{12}H_{28}NO_2Si_2$	11.74
Succinic acid 2TBDMS	289–296	0.1	15	$C_{12}H_{25}O_4Si_2$	12.04
Fumaric acid 2TBDMS	287–294	0.1	15	$C_{12}H_{23}O_4Si_2$	12.18
Serine 2TBDMS	390–396	0.2	15	$C_{17}H_{40}NO_3Si_3$	13.54
Methionine 2TBDMS	320–328	0.2	10	$C_{13}H_{30}NO_2SSi_2$	14.17
2-Oxoglutaric 1MeOX 2TBDMS	346–354	0.2	10	$C_{14}H_{28}NO_5Si_2$	14.86
Malic acid 3TBDMS	419–426	0.2	15	$C_{18}H_{39}O_5Si_3$	15.06
Aspartic acid 3TBDMS	418–425	0.2	15	$C_{18}H_{40}NO_4Si_3$	15.48
Glutamatic acid 3TBDMS	432–440	0.3	10	$C_{19}H_{42}NO_4Si_3$	16.56
Glutamine 3TBDMS	431–439	0.3	10	$C_{19}H_{43}N_2O_3Si_3$	18.22
Citric acid 4TBDMS	591–600	0.3	10	$C_{26}H_{55}O_7Si_4$	19.17
