# Supplementary material for: The role of HIF-1 in oncostatin M-dependent metabolic reprogramming of hepatic cells
Source: Cancer Metab. 2016 Feb 17;4:3. doi: 10.1186/s40170-016-0141-0 (PMC4756539; doi:10.1186/s40170-016-0141-0)
Supplement: Additional file 2 — Figure S1. Effects of OSM and hypoxia on HIF-1 α and HIF-2 α protein expression in hepatoma cells and hepatocytes. Eleven hepatoma cell lines and two immortalized hepatocyte cell lines (PH5CH8 and THLE-2) were treated with 20 ng/ml OSM (O) or grown at 1 % O2 (H) over 8 and 36 h. (A) HIF-1 α protein expression after OSM treatment represented in comparison to untreated cells (Ut). (B) HIF-1 α protein expression after OSM treatment represented in comparison to cells grown under hypoxia. (C) HIF-2 α protein expression after OSM treatment represented in comparison to untreated cells. (D) HIF-2 α protein expression after OSM treatment represented in comparison to cells grown under hypoxia. (E) HIF-1 α and HIF-2 α protein induction in non-neoplastic THLE-2 hepatocytes after OSM or hypoxia or a combinatorial treatment. (PDF 655 kb) [file 40170_2016_141_MOESM2_ESM.pdf]

Additional file 2: Figure S1.

A

OSM compared to normoxia

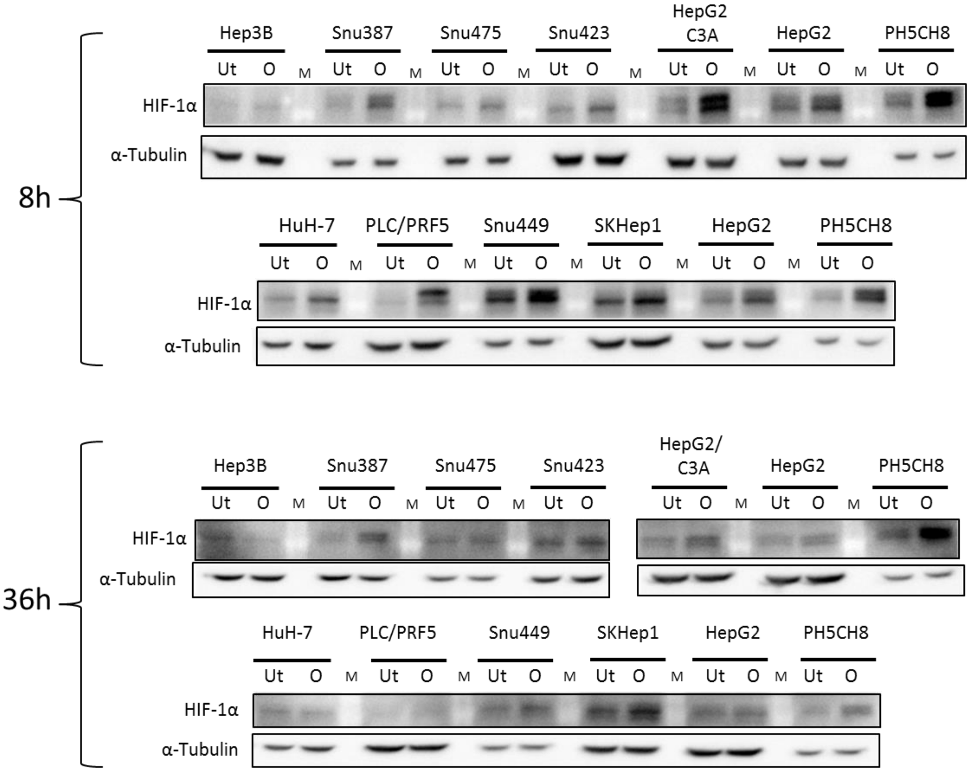

B

OSM compared to hypoxia

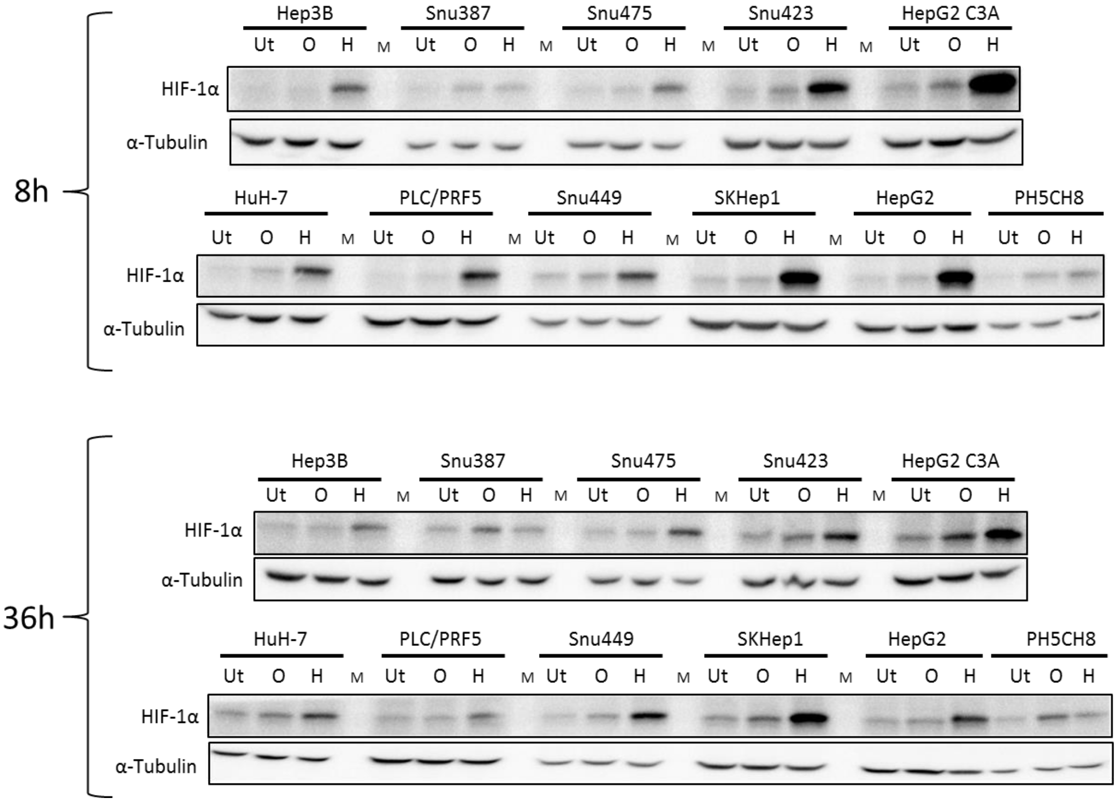

C

OSM compared to normoxia

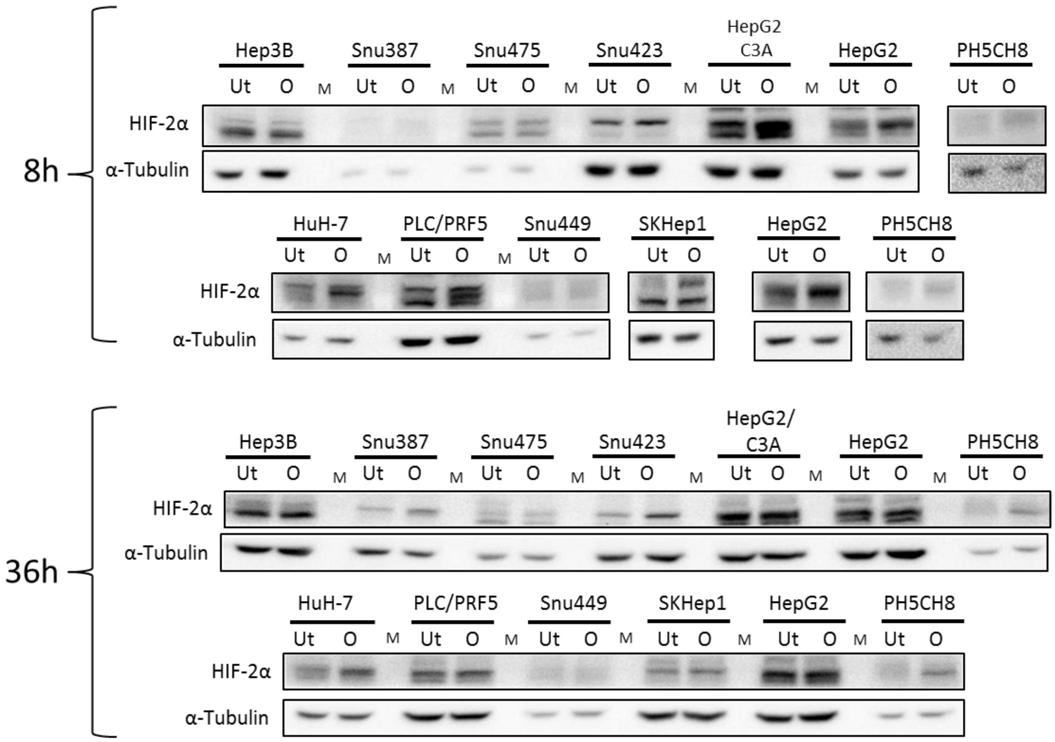

D

OSM compared to hypoxia

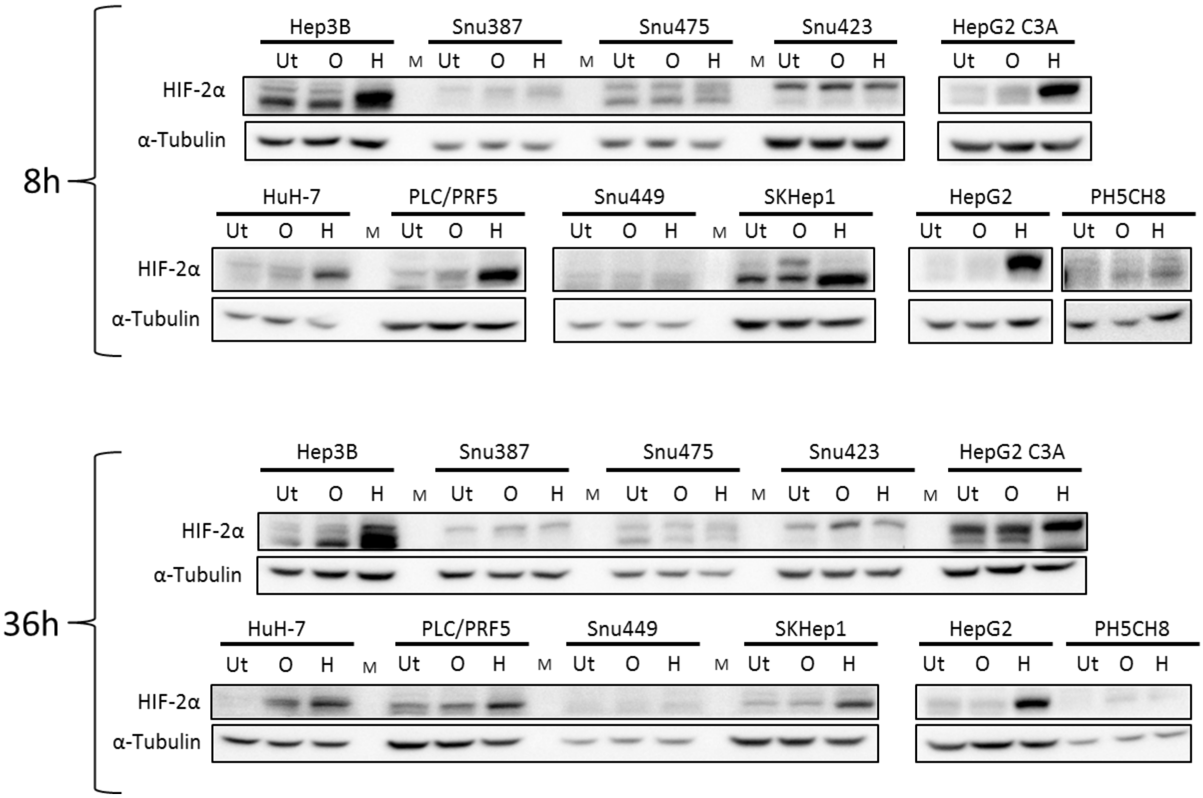

E

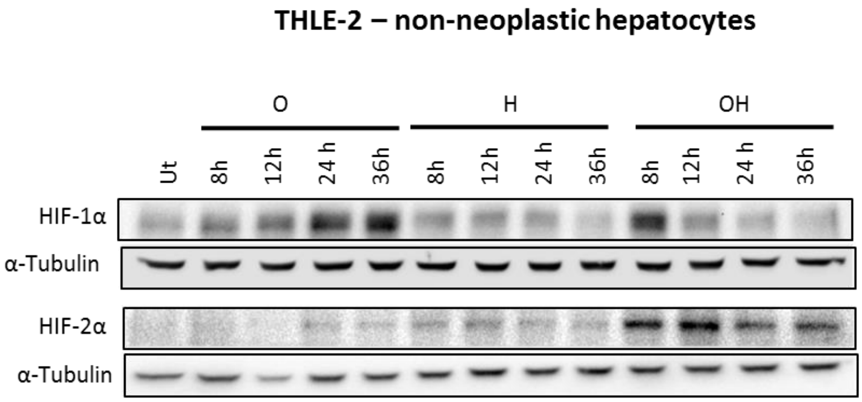
THLE-2 – non-neoplastic hepatocytes
O
H
OH
Ut
8h
12h
24 h
36h
8h
12h
24 h
36h
8h
12h
24 h
36h
HIF-1α
α-Tubulin
HIF-2α
α-Tubulin
