# Supplementary figures and images for: The role of HIF-1 in oncostatin M-dependent metabolic reprogramming of hepatic cells
Source: Cancer Metab. 2016 Feb 17;4:3. doi: 10.1186/s40170-016-0141-0 (PMC4756539; doi:10.1186/s40170-016-0141-0)

# Additional file 3: Figure S2

A

Glucose uptake

Lactate secretion

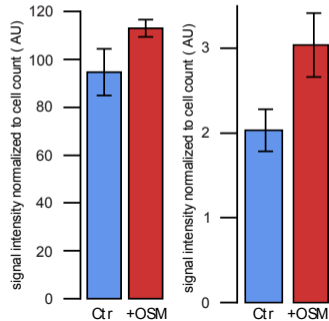

B

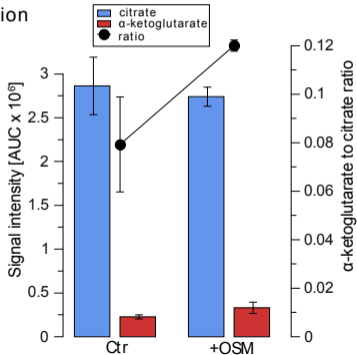

Supplement: Additional file 3 — Figure S2. (A) Glucose uptake and lactate secretion rates in PH5CH8 immortalized hepatocytes in response to a 36 h treatment with 50 ng/mL OSM. Uptake and secretion rates were determined by GC/MS. (B) α-ketoglutarate to citrate ratios in PH5CH8 cells treated with 50ng/mL of OSM for 36 h in comparison to the respective untreated control. (PDF 42.2 kb) [file 40170_2016_141_MOESM3_ESM.pdf]

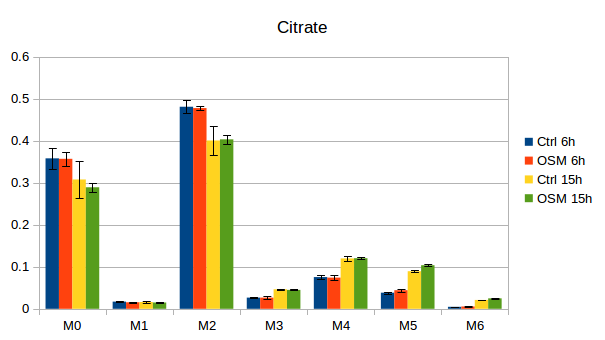

Supplement: Additional file 7 — Figure S4. Mass isotopomer distributions (MIDs) from [13C6]glucose in HepG2 human hepatoma cells treated for 6 and 15 h with 50 ng/mL OSM or left untreated. MIDs were corrected for natural isotope abundance. (PNG 14.8 kb) [file 40170_2016_141_MOESM7_ESM.png]
