# Supplementary material for: The role of HIF-1 in oncostatin M-dependent metabolic reprogramming of hepatic cells
Source: Cancer Metab. 2016 Feb 17;4:3. doi: 10.1186/s40170-016-0141-0 (PMC4756539; doi:10.1186/s40170-016-0141-0)
Supplement: Additional file 4 — Table S2. Mass isotopomer distributions (MIDs) from [13C6]glucose in PH5CH8 immortalized human hepatocytes treated for 36 h with 50 ng/mL OSM or left untreated. MIDs were corrected for natural isotope abundance. (PDF 94.2 kb) [file 40170_2016_141_MOESM4_ESM.pdf]

Additional file 4: Table S2.

Metabolite		Ctr		Ctr + OSM	
		Value	Standard deviation	Value	Standard deviation
Aspartic acid	M0	0.86	1.74E-003	0.90	2.24E-004
	M1	0.01	6.29E-004	0.00	1.72E-004
	M2	0.09	8.12E-004	0.06	2.93E-004
	M3	0.04	2.68E-004	0.04	1.79E-005
	M4	0.01	1.81E-004	0.00	6.05E-005
Lactic acid	M0	0.07	1.07E-002	0.09	3.10E-002
	M1	0.00	1.67E-004	0.00	2.36E-004
	M2	0.03	3.57E-004	0.03	1.15E-003
	M3	0.90	1.02E-002	0.88	2.99E-002
Alanine	M0	0.14	3.16E-003	0.14	5.04E-003
	M1	0.01	5.72E-005	0.00	8.85E-005
	M2	0.03	4.01E-005	0.03	4.35E-004
	M3	0.83	2.93E-003	0.83	4.54E-003
Glutamic acid	M0	0.85	7.89E-003	0.89	2.05E-003
	M1	0.01	3.95E-004	0.01	1.74E-004
	M2	0.11	1.29E-003	0.07	1.28E-003
	M3	0.01	9.13E-005	0.01	2.53E-004
	M4	0.01	1.63E-004	0.01	2.47E-004
	M5	0.00	9.33E-003	0.00	1.15E-004
Fumaric acid	M0	0.86	9.93E-004	0.90	2.49E-003
	M1	0.01	2.81E-003	0.00	3.11E-003

Continued on next page

	M2	0.09	1.48E-003	0.05	3.56E-004
	M3	0.04	5.57E-004	0.04	4.77E-004
	M4	0.01	3.10E-004	0.00	1.39E-004
α-ketoglutarate	M0	0.89	1.29E-002	0.94	3.25E-003
	M1	0.00	3.98E-003	0.00	1.14E-003
	M2	0.10	1.00E-003	0.07	2.07E-003
	M3	0.01	1.53E-003	0.01	3.10E-004
	M4	-0.02	2.27E-004	-0.01	2.89E-004
	M5	0.01	1.40E-002	0.00	3.63E-007
Citric acid	M0	0.31	7.77E-003	0.46	3.45E-003
	M1	0.02	1.93E-004	0.01	6.22E-004
	M2	0.52	6.93E-003	0.40	1.14E-003
	M3	0.02	1.41E-003	0.02	3.61E-004
	M4	0.09	1.03E-003	0.07	5.86E-004
	M5	0.04	8.40E-004	0.04	1.39E-004
	M6	0.01	4.13E-004	0.01	5.87E-004
