# Supplementary material for: The role of HIF-1 in oncostatin M-dependent metabolic reprogramming of hepatic cells
Source: Cancer Metab. 2016 Feb 17;4:3. doi: 10.1186/s40170-016-0141-0 (PMC4756539; doi:10.1186/s40170-016-0141-0)
Supplement: Additional file 5 — Table S3. Mass isotopomer distributions (MIDs) from [13C5]glutamine in PH5CH8 immortalized human hepatocytes treated for 36 h with 50 ng/mL OSM or left untreated. MIDs were corrected for natural isotope abundance. (PDF 94.2 kb) [file 40170_2016_141_MOESM5_ESM.pdf]

Additional file 5: Table S3.

Metabolite		Ctr Value	Ctr Standard deviation	Ctr + OSM Value	Ctr + OSM Standard deviation
Aspartic acid	M0	0.05	4.78E-004	0.06	1.20E-003
	M1	0.03	1.11E-003	0.02	7.21E-004
	M2	0.11	1.97E-003	0.08	1.36E-003
	M3	0.18	8.73E-004	0.25	1.09E-003
	M4	0.64	2.83E-003	0.59	4.60E-003
Lactic acid	M0	0.98	3.21E-004	0.98	9.90E-004
	M1	0.00	9.15E-005	0.00	2.90E-004
	M2	0.00	1.27E-004	0.00	1.44E-004
	M3	0.02	5.10E-004	0.01	7.55E-004
Alanine	M0	0.89	2.05E-003	0.91	2.13E-003
	M1	0.00	1.19E-004	0.00	7.62E-004
	M2	0.02	9.44E-005	0.02	3.16E-004
	M3	0.09	1.87E-003	0.08	1.58E-003
Glutamic acid	M0	0.00	5.98E-004	0.00	2.98E-005
	M1	0.00	7.96E-004	0.00	1.90E-005
	M2	0.00	1.63E-004	0.00	1.60E-005
	M3	0.00	5.86E-005	0.00	1.02E-004
	M4	0.04	1.13E-004	0.04	2.13E-004
	M5	0.96	7.37E-004	0.97	3.41E-004
Fumaric acid	M0	0.06	6.17E-004	0.07	1.92E-003
	M1	0.02	9.40E-004	0.02	3.30E-004

Continued on next page

Additional file 5: Table S3. continued from previous page

	M2	0.10	2.70E-003	0.08	1.36E-003
	M3	0.18	6.00E-004	0.25	1.40E-003
	M4	0.64	3.02E-003	0.59	3.24E-003
α-ketoglutarate	M0	0.03	1.22E-002	0.03	1.08E-002
	M1	0.01	4.78E-003	0.01	3.20E-003
	M2	0.02	1.78E-003	0.01	1.37E-002
	M3	0.10	4.97E-003	0.07	8.13E-003
	M4	0.03	1.65E-003	0.03	3.21E-003
	M5	0.82	1.10E-002	0.89	4.38E-002
Citric acid	M0	0.08	3.65E-003	0.08	2.19E-003
	M1	0.04	1.68E-003	0.03	4.05E-005
	M2	0.11	3.59E-003	0.10	1.93E-003
	M3	0.12	1.48E-003	0.12	1.96E-003
	M4	0.43	6.17E-003	0.33	2.34E-003
	M5	0.19	1.07E-002	0.32	4.30E-003
	M6	0.03	1.21E-003	0.02	7.37E-004
