# Supplementary material for: The role of HIF-1 in oncostatin M-dependent metabolic reprogramming of hepatic cells
Source: Cancer Metab. 2016 Feb 17;4:3. doi: 10.1186/s40170-016-0141-0 (PMC4756539; doi:10.1186/s40170-016-0141-0)
Supplement: Additional file 6 — Figure S3. Response of PH5CH1 and PH5CH7 non-neoplastic hepatocytes to OSM, hypoxia, and a combination of both stimuli. PH5CH1 and PH5CH7 cells were treated with OSM (50 ng/ml), hypoxia (1 % O2), or a combinatorial treatment for the indicated periods of time. (A, D) Quantitative RT-PCR of PDP2, PDK1, and HIF-1 α mRNA. Fold changes were calculated relative to the untreated control. (B, E) Western blot analysis for HIF-1 α, HIF-2 α, PDK1, and all three PDH phosphorylation sites (S232, S293, S300). Vinculin and α-tubulin were used as loading controls, and one representative blot is shown. (C, F) Quantification of PDK1 protein levels. (PDF 428 kb) [file 40170_2016_141_MOESM6_ESM.pdf]

Additional file 6: Figure S3.

A

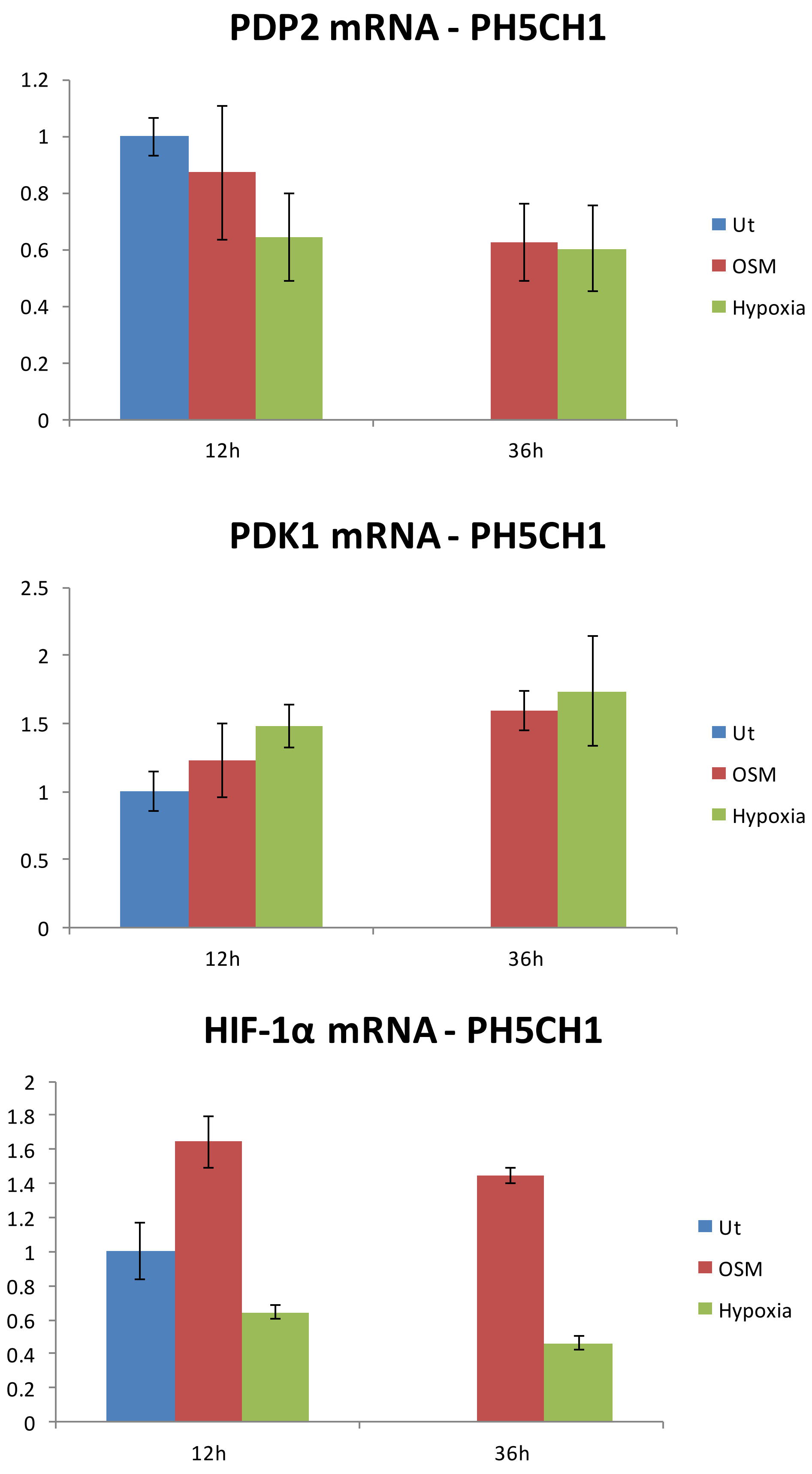

PH5CH1

B

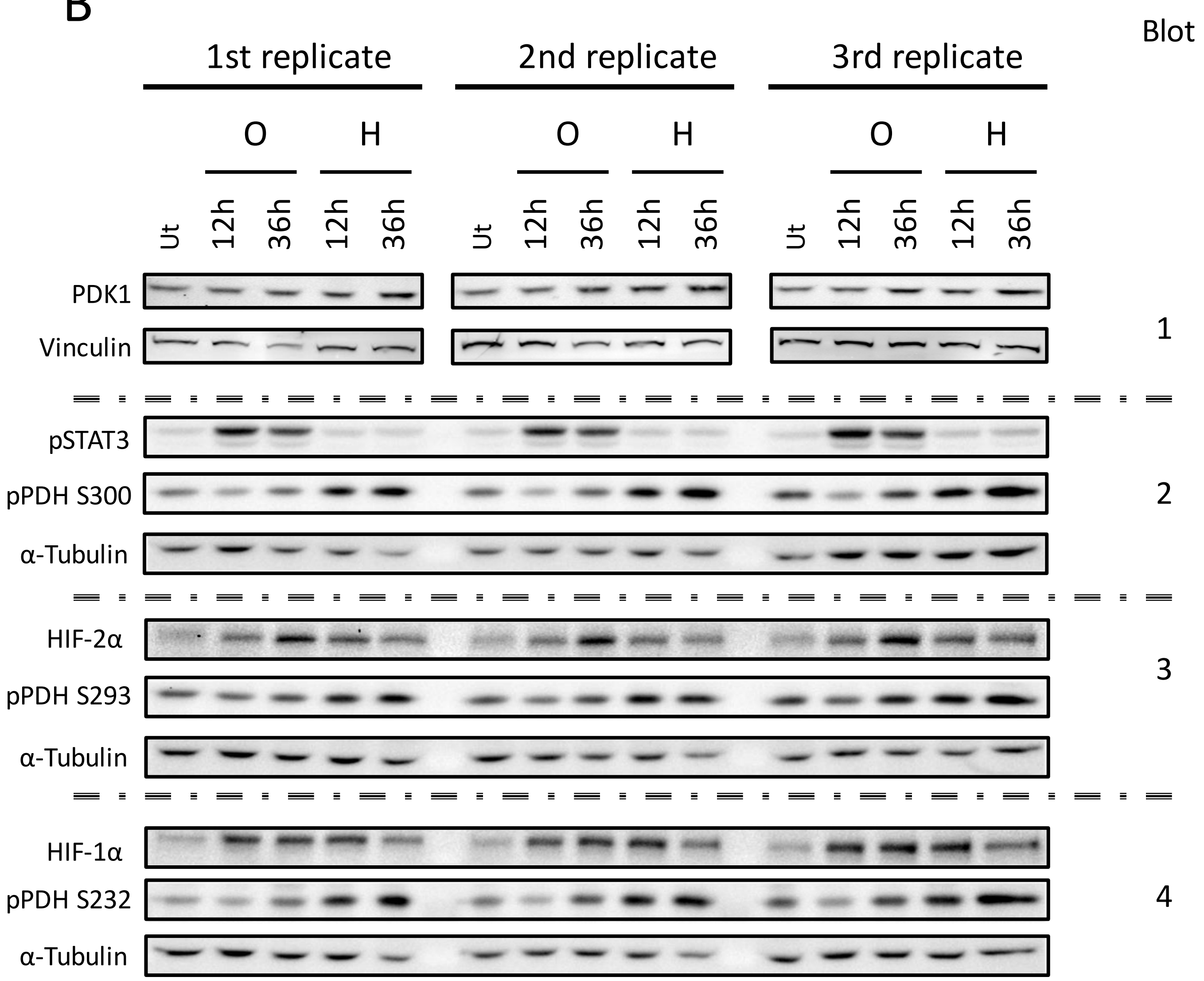

Blot
1st replicate
2nd replicate
3rd replicate
O
H
Ut
12h
36h
PDK1
Vinculin
1
pSTAT3
pPDH S300
α-Tubulin
2
HIF-2α
pPDH S293
α-Tubulin
3
HIF-1α
pPDH S232
α-Tubulin
4

C

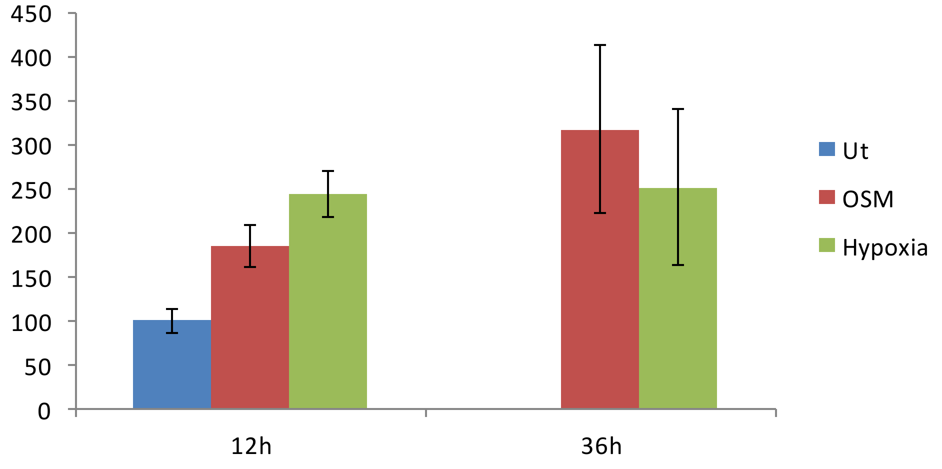

PDK1 protein level - PH5CH1
450
400
350
300
250
200
150
100
50
0
12h
36h
Ut
OSM
Hypoxia

D

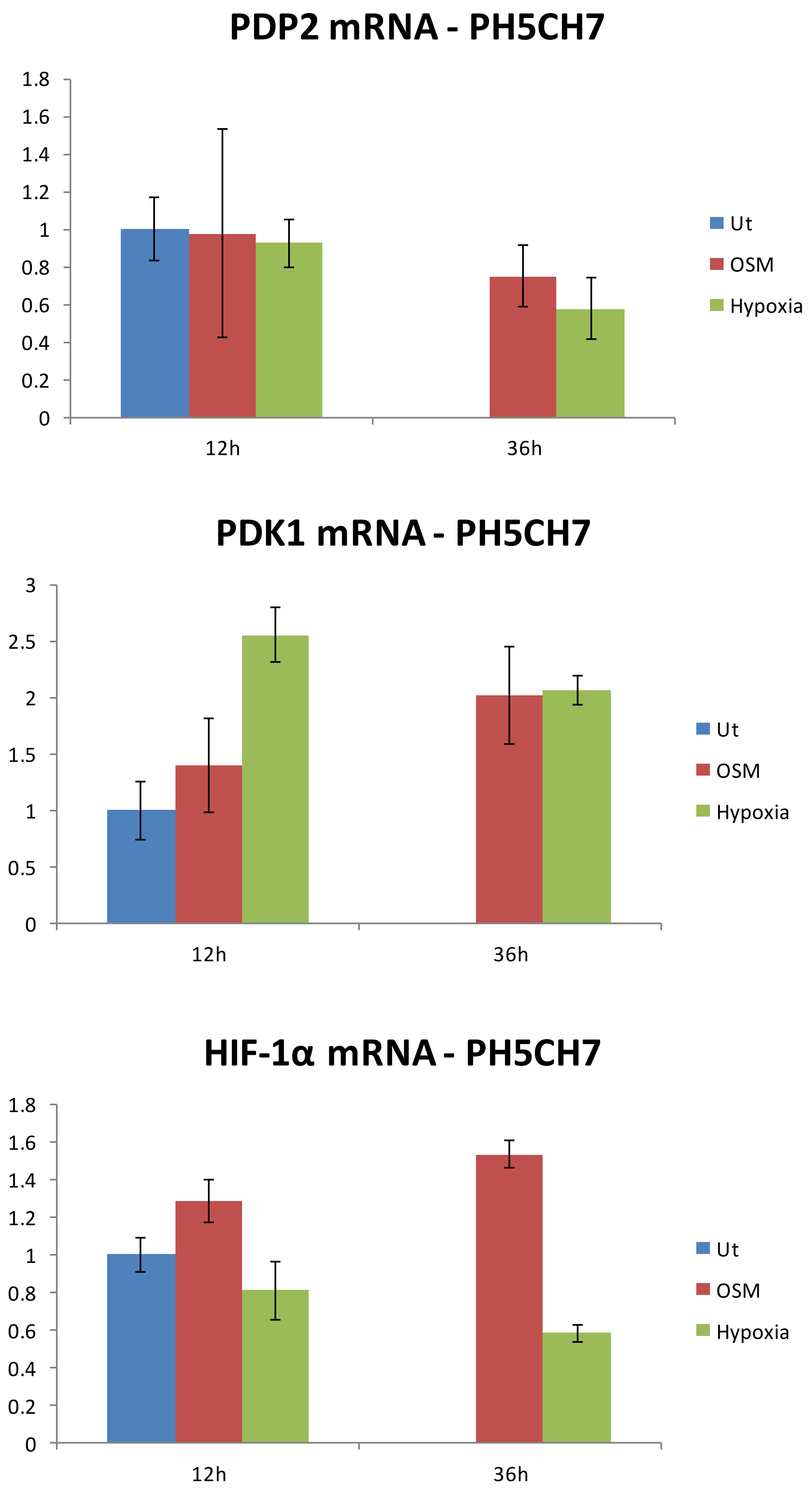
PDP2 mRNA - PH5CH7
1.8
1.6
1.4
1.2
1
0.8
0.6
0.4
0.2
0
12h
36h
Ut
OSM
Hypoxia
PDK1 mRNA - PH5CH7
3
2.5
2
1.5
1
0.5
0
12h
36h
Ut
OSM
Hypoxia
HIF-1α mRNA - PH5CH7
1.8
1.6
1.4
1.2
1
0.8
0.6
0.4
0.2
0
12h
36h
Ut
OSM
Hypoxia

PH5CH7

E

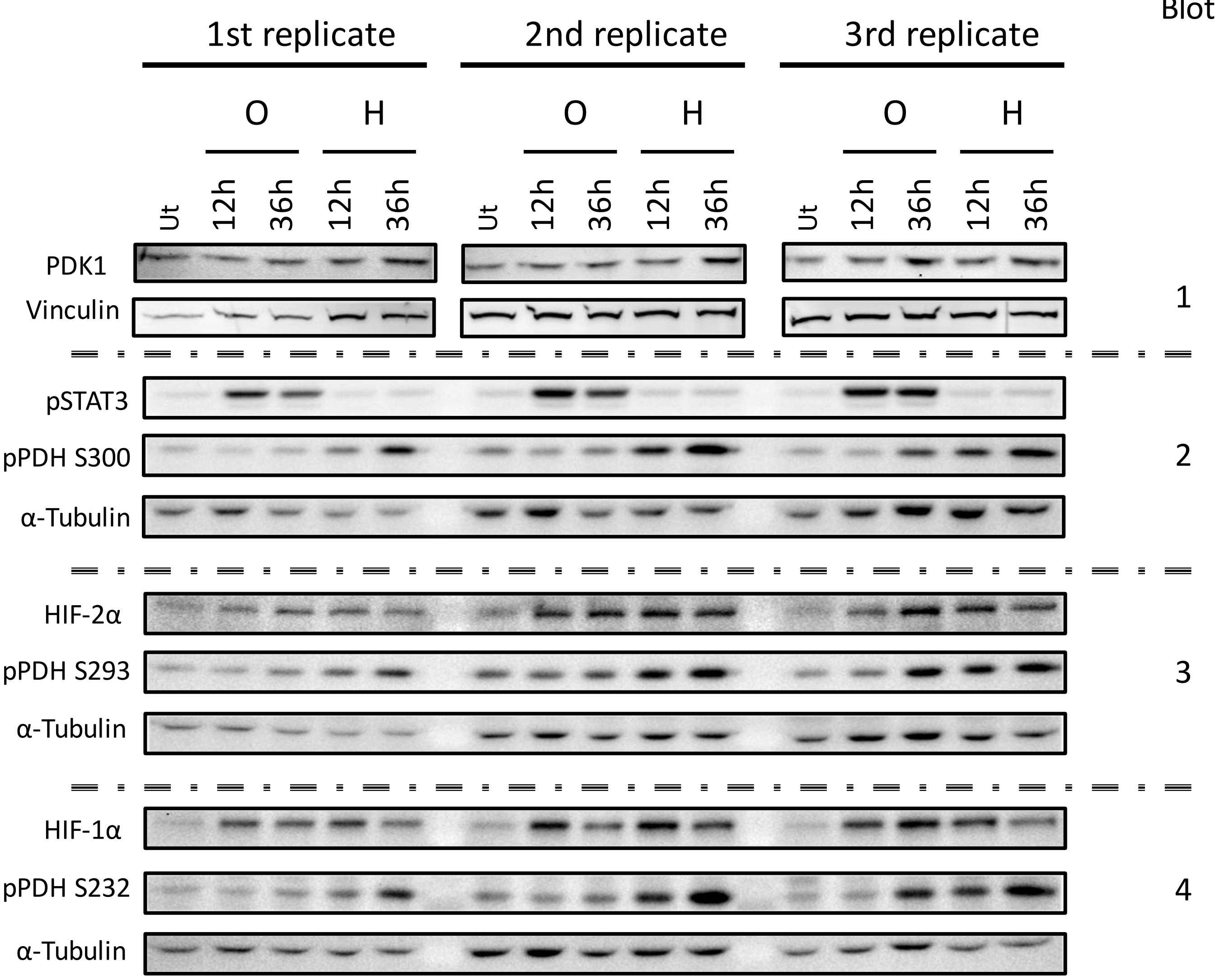

1st replicate
2nd replicate
3rd replicate
Blot
O
H
Ut
12h
36h
PDK1
Vinculin
1
pSTAT3
pPDH S300
α-Tubulin
2
HIF-2α
pPDH S293
α-Tubulin
3
HIF-1α
pPDH S232
α-Tubulin
4

F

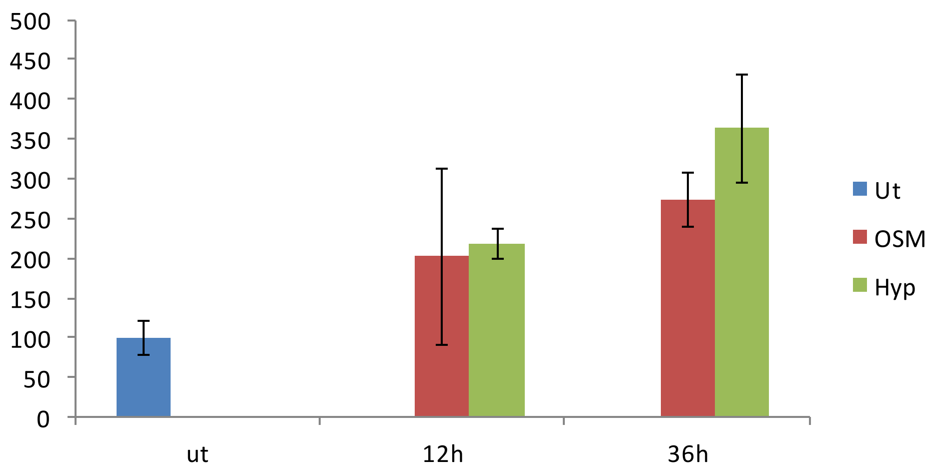

PDK1 - Protein level - PH5CH7
500
450
400
350
300
250
200
150
100
50
0
ut
12h
36h
Ut
OSM
Hyp
